# Supplementary material for: High-resolution imagery and neural networks link post-tsunami land cover changes to population health and well-being
Source: Commun Earth Environ. 2026 Mar 18;7(1):396. doi: 10.1038/s43247-026-03396-0 (PMC13149324; doi:10.1038/s43247-026-03396-0)
Supplement: Supplementary file 2 — Supplemental Information High-resolution imagery and neural networks link post-tsunami land cover changes to population health and well-being [file 43247_2026_3396_MOESM2_ESM.pdf]

Supplementary Information

High-resolution imagery and neural networks link post-tsunami land cover changes to population health and well-being

Eric Peshkin
Duke University

Elizabeth Frankenberg
University of North Carolina at Chapel Hill

Peter Katz
Duke University

Cecep Sumantri
SurveyMETER

Duncan Thomas
Duke University

Supplementary Notes

1. The Digital Globe / Maxar Imagery

In response to our application for imagery through its imagery grant program, which provided precise guidelines for the amount of data that could be requested, the DigitalGlobe Foundation (now Maxar) provided us with 11 pairs of panchromatic and multispectral images from the Quickbird satellite. The images spanned a 4.8 year period and covered a region at the northern end of the island of Sumatra. This region, which was badly damaged in the 2004 Indian Ocean tsunami, includes the city of Banda Aceh (the capital of Aceh Province) as well as less densely populated coast and rural areas. The raw panchromatic imagery is at 61 cm resolution, while the multispectral imagery is at 2.44 meter resolution. The images are georeferenced to facilitate linking them to their positions on the globe.

Each of the 11 pairs of image tiles captures Aceh at a different point in time. The earliest image is from June 23, 2004. The tsunami occurred six months later, on December 26, 2004. The other images are all from after the tsunami and were captured on December 28, 2004; December 30, 2004; January 22, 2005; August 6, 2005; January 28, 2006; May 16, 2006; July 30, 2007; November 4, 2008; February 10, 2009; and February 23, 2009. The tiles vary with respect to the geographic area captured, so some areas are represented in the imagery more frequently than others. In addition, cloud cover obscures the land surface for some areas in some years. We used Gram-Schmidt pansharpening to combine corresponding multispectral and panchromatic images into one image with strong spatial and spectral resolution.

2. Census Data

Statistics Indonesia, the statistical agency of Indonesia, conducts decennial censuses. In 2005 Statistics Indonesia also conducted a special census of Aceh and Nias, the areas most affected by the disaster. We obtained the 2005 and 2010 census data from Statistics Indonesia and use them to provide population counts in each year for the 164 administrative areas in the region for which satellite imagery is available.

3. The Study of the Tsunami Aftermath and Recovery (STAR) data

The Study of the Tsunami Aftermath and Recovery (STAR) is a longitudinal survey of individuals, households, and communities in the tsunami-affected provinces of Aceh and North Sumatra, Indonesia. The baseline sample comes from the 2004 National Socioeconomic Survey (SUSENAS), an annual survey conducted by Statistics Indonesia 10 months before the tsunami. SUSENAS is representative of the population at the district (*kabupaten*) level (BPS 2019). Within kabupaten, random selection was used first to select enumeration areas (clusters of households defined by Statistics Indonesia) and second, within each enumeration area, to select 16 households for interview. Household members answered questions with respect to household composition and survival of household members in 2004, as well as about many other socioeconomic attributes measured at both the household and the individual level and about physical and psychosocial health.

The STAR survey targeted thirteen districts in Aceh and North Sumatra, all with coastlines potentially vulnerable to inundation from the tsunami. All SUSENAS enumeration areas (EAs) within these districts were eligible for the STAR survey in 2005. Together the EAs capture wide variation in the severity of the disaster.

The first wave of STAR sought to re-interview all individuals who were members of households interviewed in the selected districts as part of SUSENAS in 2004. STAR1 targeted 7,157 households containing 28,376 individuals. Wave 1 fieldwork began five months post- tsunami

(May 2005) and lasted for 12 months. Four additional follow-ups were conducted annually, concluding in November 2010. A 10-year follow-up took place between September 2014 and June 2016 and a 15-year follow-up took place between 2019 and 2022. Across all survey rounds we succeeded in obtaining at least one interview for over 95% of target respondents. For this paper we use the STAR data from Waves 1 and 5, collected for households who, in 2004, were in one of the 43 enumeration areas in the region for which the satellite imagery is available.

Supplementary Methods

1. Labelling

We developed definitions and visual examples of the eight classes of landcover on which we focus (agriculture, water, buildings, roads, foundation, rubble, beach, and cloud). We trained individuals in the United States and Indonesia to hand label the imagery data. Images assigned for hand labelling were selected from throughout the 4.8 year time period and covered many diverse examples across all classes we sought to distinguish. We regularly reviewed labelled images and discussed changes with the labelling team to facilitate consistency of labelling. The classes most difficult for the labelers to distinguish were rubble and foundation. These classes were defined so that their union was consistent over time (allowing them to be combined for the purpose of linkage analysis), but the definition of each relative to the other was updated as imagery was labeled to improve consistency as necessary, given that these classes capture overlapping concepts which can be difficult to crisply, visually demarcate.

2. Training and Hyperparameter Optimization

The hyperparameters that were optimized and the initial search ranges are provided in Supplementary Table 2, along with the optimal value of each as determined for the primary network, which was trained without removing any training or validation data via the held-out procedure (i.e., without removing any data potentially overlapping with one of the STAR enumeration areas).

3. Performance

In the primary network, each class, apart from rubble, cloud, and other, achieves a higher score on recall than on precision (Table 4), suggesting that the network was better at avoiding false negatives (incorrectly excluding a pixel from the class to which it belongs) than avoiding false positives (labelling a pixel as part of a class to which it does not belong).

Supplementary Tables 3 and 4 present the confusion matrices for the primary network evaluated against the labeled test set in two different ways: first by comparing the test set labels to segmentations produced from the test set imagery when input into the network in individual 250x250 pixel slices, without the benefit of the voter procedure applied at segmentation, and second by comparing each region represented in the labeled test set to the corresponding labeled location in our large-scale segmentation output (which such segmentations benefit from the voting procedure). The mild increase in each class's F_1 performance supports the benefit of the voting system at segmentation since the networks, taken together, generally have access to additional visual information to make the proper classification at points for which such information would otherwise be unavailable due to image boundaries.

The confusion matrix reveals other patterns among the classes, some predictable and some surprising. Predictably, water and agriculture are often confused, since it is difficult to discern, even with the help of the infrared band, flooded agriculture (which ought to be labelled “water” to

signify its destruction), aquaculture (which also appears as plots and ought to be labelled as "agriculture" to capture the fact it is productive land) and irrigated agriculture (which ought to be labelled "agriculture" since it is economically productive, unlike flooded fields in the aftermath of the tsunami). This trilemma exemplifies difficulties arising from attempting to extract important features which are visually similar but that differ in their economic implications. Rubble and foundation are difficult to distinguish for human labelers as a semantic matter, so it is not surprising that the network could not reliably distinguish between them or between them and the heterogenous "other" class. While distinguishing the nature of destruction at such a granular level could be useful, it is complicated by the variety of forms destruction can take. That water is often confused with road may seem surprising, but is consistent with the idea that both appear as long, continuous strips that connect places (in Aceh, in addition to the ocean, rivers, canals, and ditches for irrigation and drainage are abundant). Similarly, agricultural plots, aquaculture plots, and rivers may be bounded or bordered by roads or paths, which may contribute to ambiguity and misclassifications between agriculture, water, and road.

4. Pre-Tsunami (baseline) Shares

To provide a context for the region we study, we report the distribution of pixels across landcover categories for the June 23, 2004 (pre-tsunami) image. For the region captured in this image, our CNN estimates that agriculture accounted for 43.4% of landcover, followed by buildings (8.7%), roads (4.5%), water (1.3%), and beach (0.1%). Before the tsunami levels of rubble and foundations were low (both at 0.01%). In this image clouds accounted for 3.95% of pixels.

5. Geolocation and linkage to census and survey data

The satellite imagery is georeferenced. We use ArcGIS to link the imagery to base maps of Aceh, which include the boundaries of administrative areas (villages or municipalities, depending on whether an area is classified as rural or urban by Statistics Indonesia). These linkages let us determine how the landcover of a particular location varies over time. To construct measures of landcover linked to census data, for each of the 164 administrative areas for which imagery is available we compute land cover shares within the area demarcated by the administrative boundaries. To link to the STAR survey data we define circles that are centered on the geographic center of the survey enumeration area. For each enumeration area we calculate land cover within a circle with a radius of 500m. This size covers enough land area to be substantively meaningful for a survey households while allowing us to avoid any overlap between circles of neighboring communities and to minimize differences over time in the number of pixels analyzed that arise because of image to image variation in the coverage area (the median number of pixels per circle is 3.117 million).

Supplementary Discussion

1. Independence of interview outcomes to land cover change

Fielding a census or survey in the aftermath of a disaster is complicated, and it is possible that completion rates could differ across relatively more and less damaged areas in ways that introduced bias with respect to the characteristics of the people successfully interviewed.

Focusing on the 43 STAR EAs that we analyze in this paper, in STAR1 (2005), of the 2947 individuals listed in the 2004 household rosters, we found and interviewed over 88% of the survivors. Among adults who survived both the tsunami and the subsequent five years, 93% were interviewed face to face in both STAR1 and STAR5.

To examine links between landcover change and interview outcomes, we analyze whether changes in landcover shares after the tsunami relative to just before are related to interview outcomes at the household and individual level. Holding constant land cover shares in 2004, shares in 2005 are unrelated to whether we were able to conduct a household interview in 2005. Turning to individuals who survived the tsunami, landcover shares in 2005 are also unrelated to obtaining interviews in 2005 or in 2009. Our estimates of the impacts of land cover change on individual markers of well-being do not appear to be driven by attrition that is selective on patterns of landcover change related to the tsunami.

2. Other Satellite-based Measures of Destruction and Recovery

Other analyses that link satellite imagery to data on outcomes in the aftermath of a disaster have relied on measures of nightlight intensity and change in the spectral signature of vegetation (most often relying on the normalized difference vegetation index, or NDVI). As reported in the main text, we estimated our models, replacing our measures of land cover shares constructed from our CNN segmentations with measures of nightlight intensity in 2004 and 2005 and with measures of NDVI in 2004 and 2005. Results from these analyses are displayed in Supplementary Table 1.

As discussed in the paper, the measures of NDVI operate similarly to the landcover shares but explain much less of the variation than the models with landcover. The models that include nightlights are extremely difficult to interpret. When statistically significant the signs on the coefficients are the opposite of what one would expect, and the R^2s of these models are low. These patterns may arise because our sites are close together but the resolution of the nightlight data is at best 1 km, so cross-site variation in light intensity may not differentiate damage or recovery trajectories.

We also estimate the models using a categorical measure of tsunami damage that we have used in previous work, which incorporates satellite measures of bare earth in the aftermath of the tsunami, community leaders' reports of damage, and survey supervisors' direct observations (Gray et al. 2014). This measure divides communities into three categories: heavy damage, moderate damage, and light or indirect damage. We run our models using this classification and report the results in Supplementary Table 1.

Individuals from communities in the heavy damage category are significantly less likely to survive and more likely to have been displaced in the tsunami's aftermath (the displacement result holds for those from moderately damaged communities as well). The heavy damage category is also related to lower ratings of SES. As we have written elsewhere, this measure reflects tsunami impacts that have major negative implications for well-being. Relative to our new land cover measures, however, it explains considerably less variation in these outcomes, providing further evidence that the land cover shares are highly useful in understanding how disasters affect people.

Our categorical damage measure does not reflect reconstruction after the tsunami. As part of STAR we collected extensive community-level data from village leaders and others. For the purposes of comparison to using land cover changes to understand recovery, we used this data to construct a measure indicating the community leader's view (reported in 2009) of the degree to which his or her community had recovered since the tsunami (yes, or no, versus somewhat) and we ran the models of changes with these indicators. They are unrelated to individual changes in post-traumatic stress reactivity and to changes in the ladder -- again confirming the importance and relevance of the newly developed measures of landcover change.

Supplementary References

1. BPS. 2019. “Indonesia - Survei Sosial Ekonomi Nasional 2004.” *IHSN (International Household Survey Network)*. Retrieved November 15, 2021 (https://catalog.ihsn.org/catalog/3052/study-description).

2. Gray C, Frankenberg E, Gillespie T, Sumantri C, Thomas D. Studying Displacement After a Disaster Using Large Scale Survey Methods: Sumatra After the 2004 Tsunami. Ann Assoc Am Geogr. 2014 Jan 1;104(3):594-612. doi: 10.1080/00045608.2014.892351. PMID: 24839300; PMCID: PMC4019446.

SI Table 1 Results for NDVI, Nightlighs, and Damage Measures

		Individual Outcomes in 2005*				Change: 2009-2005		
		(1)	(2)	(3)	(4)		(5)	(6)
		Killed in tsu.	Displaced	Post Traumatic Stress Reactivity	SES ladder Post-Pre		Post Traumatic Stress Reactivity	SES ladder 2009-2005
NDVI	2005	-1.34	-1.73	-5.26	1.09	Difference in NDVI	-3.09	0.41
		[0.21]	[0.24]	[1.66]	[0.31]		[1.07]	[0.19]
	2004	0.26	0.46	2.53	-0.20			
		[0.33]	[0.26]	[1.91]	[0.35]			
	Constant	0.60	0.92	7.85	-0.57		-4.80	0.57
		[0.12]	[0.07]	[0.79]	[0.12]		[0.57]	[0.16]
	Observations	2,947	2,065	1,109	1,109		1,109	1,109
	R-squared	0.313	0.255	0.054	0.077		0.027	0.014
Nighttime Lights	2005	0.02	-0.00	0.13	-0.01	Difference in Lights	0.05	0.00
		[0.02]	[0.01]	[0.09]	[0.01]		[0.02]	[0.00]
	2004	-0.01	0.00	-0.14	0.01			
		[0.01]	[0.01]	[0.06]	[0.01]			
	Constant	0.26	0.46	7.88	-0.30		-5.49	0.51
		[0.14]	[0.11]	[0.64]	[0.13]		[0.73]	[0.21]
	Observations	2,947	2,065	1,109	1,109		1,109	1,109
	R-squared	0.078	0.056	0.061	0.024		0.020	0.008
STAR Community-Level Damage	Heavy	0.30	0.69	0.65	-0.26	Village Recovered	-0.69	0.01
		[0.10]	[0.08]	[1.30]	[0.06]		[0.66]	[0.15]
	Moderate	0.02	0.30	-0.05	0.02	Village Not Recovered	-1.17	0.13
		[0.05]	[0.07]	[1.34]	[0.04]		[1.18]	[0.20]
	Constant	0.10	0.05	9.86	-0.11		0.52	-3.92
		[0.09]	[0.18]	[1.51]	[0.09]		[0.26]	[0.71]
	Observations	2,947	2,065	1,109	1,109		1,109	1,109
	R-squared	0.141	0.196	0.040	0.057		0.008	0.017
	p(F)	0.00125	7.45e-10	0.272	2.59e-05		0.623	0.513

SI Table 2: Hyperparameter optimation procedure initial search ranges and results for the network trained on the entire training and validation subset

Hyperparameter	Initial Range, Type	Optimal Value (Primary Net)
Initial Learn Rate	[0.0001,0.1], real*	0.0394
Momentum	[0.60,0.99], real	0.8504
Weight Decay	[1e-10,1e-2], real*	1.07E-10
Minibatch Size	[4,20], integer	14
Number of Epochs	[100,200], integer	166
Activation Function	[ReLU, GeLU], discrete	GeLU
ASPP Dilation Factors	[Small, Medium, Standard], discrete**	Standard

*Log-spaced variable **Small = [1,3,6,9], Medium = [1,6,12,18], Standard = [1,12,24,30]

SI Table 3. Confusion matrix for the primary network trained on the complete training and validation subset

	250x250 Testing Set								
	Agriculture	Water	Rubble	Foundation	Beach	Cloud	Road	Building	Other
Agriculture	13,855,114	1,768,853	240	4,596	13,660	19,419	344,898	57,414	1,027,834
Water	1,151,371	7,766,763	21,597	12,903	19,637	1,515	127,898	14,531	1,089,016
Rubble	7,261	29,473	442,289	112,419	90	16	7,110	38,934	632,751
Foundation	3,400	158	3,544	62,431	0	160	722	84,976	69,690
Beach	0	24,208	0	0	496,174	0	146	15	40,043
Cloud	266,981	16,635	0	1,818	31,903	4,705,513	65,861	96,698	327,577
Road	149,668	112,977	6,102	672	2,428	15,135	2,166,407	44,261	503,305
Building	9,371	11,643	9,206	15,792	1,272	26,630	33,714	5,430,590	391,091
Other	5,038,364	805,212	541,419	166,546	126,428	127,048	1,593,797	1,975,867	31,828,800

Table created by comparing the testing set labels to 250x250 pixel segementations of the testing set imagery produced by the primary network, without the benefit of the voting procedure applied at segmentation

SI Table 4. Confusion matrix for the primary network trained on the complete training and validation subset

	Segmentation Set								
	Agriculture	Water	Rubble	Foundation	Beach	Cloud	Road	Building	Other
Agriculture	14,137,478	1,594,129	220	4,601	5,171	15,779	333,809	47,776	953,065
Water	1,077,302	7,800,420	18,745	11,029	17,423	368	127,699	13,213	1,139,032
Rubble	4,770	29,320	444,677	106,323	10	165	5,692	36,312	643,074
Foundation	2,664	96	3,054	64,418	7	65	963	84,401	69,413
Beach	10	24,931	0	0	497,037	0	69	0	38,539
Cloud	260,728	22,114	0	1,086	16,443	4,738,865	55,430	91,412	326,908
Road	144,374	109,788	5,627	519	4,590	15,537	2,192,039	38,340	490,141
Building	8,834	11,035	8,159	16,611	545	22,045	22,174	5,446,361	393,545
Other	4,704,443	745,801	509,504	148,765	114,288	120,243	1,507,409	1,917,045	32,435,983

Table created by comparing the testing set labels to the corresponding regions in the large-scale segmentations, with the benefit of the voting procedure applied at segmentation.
